# Supplementary material for: Causal interplay between lactose intolerance and gut microbiota: a combined bidirectional Mendelian randomization and in vivo validation study
Source: Front Nutr. 2026 Jun 1;13:1803337. doi: 10.3389/fnut.2026.1803337 (PMC13265576; doi:10.3389/fnut.2026.1803337)
Supplement: Supplementary file 1 [file Data_Sheet_1.zip › supplementary materials/Forward/leave one out cross validation/ebi-a-GCST90027678.finngen_R12_E4_LACTONAS.pdf]

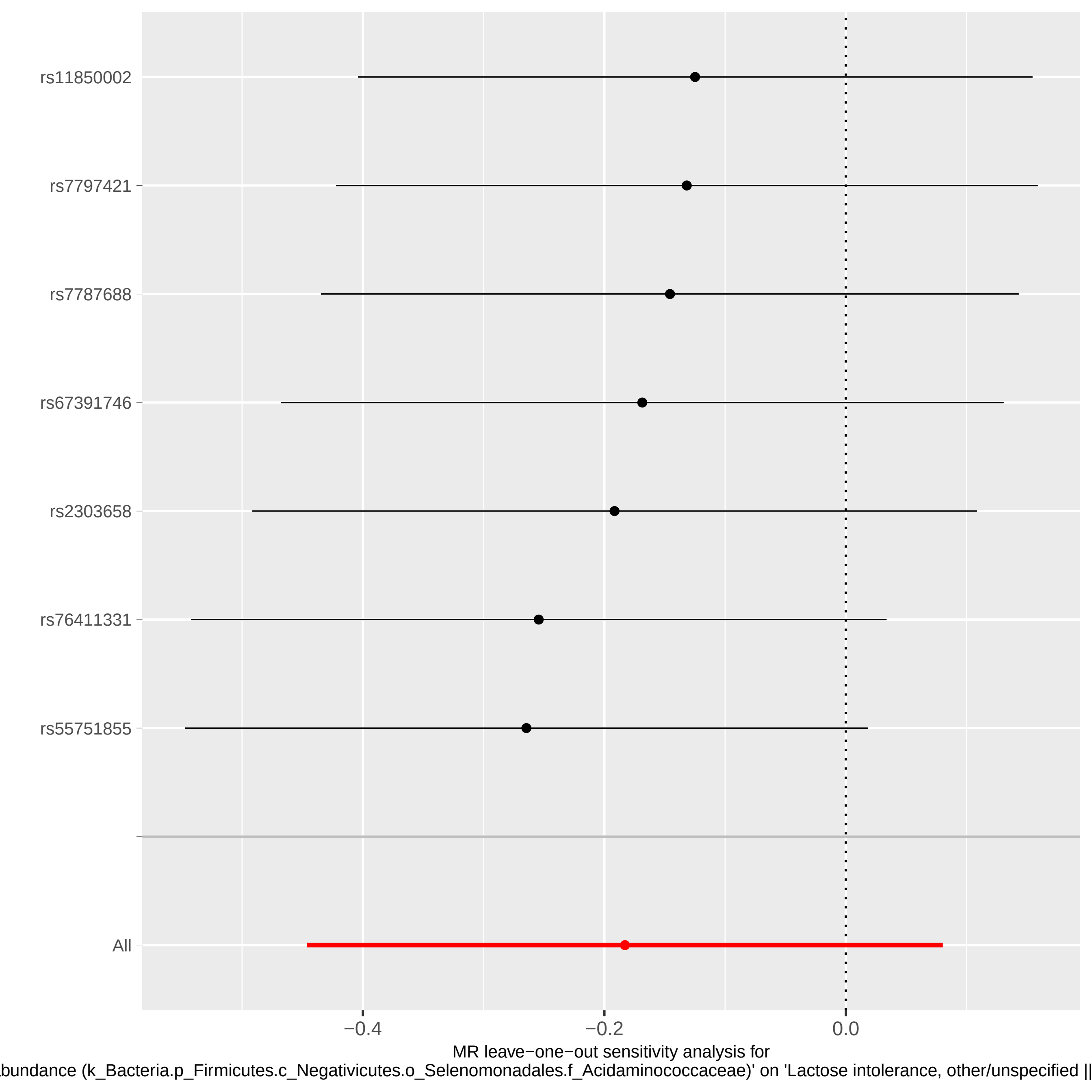
rs11850002
rs7797421
rs7787688
rs67391746
rs2303658
rs76411331
rs55751855
All
−0.4
−0.2
0.0
MR leave−one−out sensitivity analysis for
bundance (k_Bacteria.p_Firmicutes.c_Negativicutes.o_Selenomonadales.f_Acidaminococcaceae)' on 'Lactose intolerance, other/unspecified ||
